# Greek Yogurt and 12 Weeks of Exercise Training on Strength, Muscle Thickness and Body Composition in Lean, Untrained, University-Aged Males

**DOI:** 10.3389/fnut.2019.00055

**Published:** 2019-04-30

**Authors:** Aaron Bridge, Joseph Brown, Hayden Snider, Matthew Nasato, Wendy E. Ward, Brian D. Roy, Andrea R. Josse

**Affiliations:** ^1^Department of Kinesiology, Faculty of Applied Health Sciences, Brock University, St. Catharines, ON, Canada; ^2^School of Kinesiology and Health Science, Faculty of Health, York University, Toronto, ON, Canada; ^3^Centre for Bone and Muscle Health, Faculty of Applied Health Sciences, Brock University, St. Catharines, ON, Canada

**Keywords:** Greek yogurt, muscular strength, body composition, young males, muscle thickness, protein nutrition, intervention study, resistance training program

## Abstract

Milk and/or whey protein plus resistance exercise (RT) increase strength and muscle size, and optimize body composition in adult males and females. Greek yogurt (GY) contains similar muscle-supporting nutrients as milk yet it is different in several ways including being a semi-solid food, containing bacterial cultures and having a higher protein content (mostly casein) per serving. GY has yet to be investigated in the context of a RT program. The purpose of this study was to assess the effects of GY consumption plus RT on strength, muscle thickness and body composition in lean, untrained, university-aged males. Thirty untrained, university-aged (20.6 ± 2.2 years) males were randomized to 2 groups (*n* = 15/group): fat-free, plain GY or a Placebo Pudding (PP; isoenergetic carbohydrate-based pudding) and underwent a combined RT/plyometric training program 3 days/week for 12 weeks. They consumed either GY (20 g protein/dose) or PP (0 g protein/dose) daily, 3 times on training days and 2 times on non-training days. After 12 weeks, both groups significantly increased strength, muscle thickness and fat-free mass (FFM) (*p* < 0.05). The GY group gained more total strength (GY; 98 ± 37 kg, PP; 57 ± 15 kg), more biceps brachii muscular thickness (GY; 0.46 ± 0.3 cm, PP; 0.12 ± 0.2 cm), more FFM (GY; 2.4 ± 1.5 kg, PP; 1.3 ± 1.3 kg), and reduced % body fat (GY; −1.1 ± 2.2%, PP; 0.1 ± 2.6%) than PP group (*p* < 0.05 expressed as absolute change). Thus, consumption of GY during a training program resulted in improved strength, muscle thickness and body composition over a carbohydrate-based placebo. Given the results of our study, the general benefits of consuming GY and its distinctiveness from milk, GY can be a plausible, post-exercise, nutrient-rich alternative for positive strength, muscle, and body composition adaptations.

## Introduction

The use of protein supplements to facilitate resistance training (RT) adaptations has long been documented in human populations (1–3). Dairy protein, which is comprised mostly of casein and whey, is a beneficial muscle building protein due to the complete essential amino acid (AA) profile and adequate leucine levels, which is primarily responsible for muscle protein synthesis (MPS) (4–6). Dairy products may also promote fat loss, possibly due to their increased content of bioavailable calcium (7), and effect on appetite suppression (8). Whey, which is a rapidly absorbed protein, is able to provide essential AAs to the exercised muscle to begin MPS soon after consumption (1, 9). Casein, which constitutes 80% of dairy protein, is a more slowly absorbed protein, and is able to prolong elevated levels of plasma-AAs and enhance whole-body protein turnover (10). This unique characteristic of casein protein may act to attenuate muscle protein breakdown (MPB), however further research is required to specifically determine this. Nevertheless, both proteins allow for a net positive protein balance (or a less negative balance) over a prolonged period time (11). Because of this (and other important features), dairy protein, and especially milk, which has garnered the majority of research, has been shown to be an effective beverage for facilitating adaptations to RT (12, 13). This poses the question; would other dairy products elicit the same positive adaptations to RT as milk?

There is strong support for the use of isolated protein supplements, such as whey for increasing strength, muscle size, and lean mass while undergoing RT (1, 9, 14). However, research regarding whole food protein-sources is limited. It is important to study whole-food protein sources as they contain additional components such as micronutrients, antioxidants, and bacterial cultures that are beneficial to overall health, and that may improve the digestibility and absorption of nutrients such as protein from the food (15). Moreover, whole food products are more accessible and are consumed more readily by the population. In terms of milk research, Hartman et al. (12) in males, and Josse et al. (13) in females, have shown that milk consumption following RT for 12 weeks was able to produce significant strength and body composition improvements compared to isoenergetic carbohydrate (CHO)-based placebos (12, 16). However, research by Rankin et al. (17), found no benefit of chocolate milk post-RT on these outcomes compared to CHO (17). Similar to milk, GY contains important nutrients for musculoskeletal health such as calcium, phosphorus and protein, however the consistency and composition of GY is different. GY possesses unique properties including its solidity and the provision of bacterial cultures, that may provide additional health benefits (8). Solid foods are more satiating than liquid foods and can delay gastric emptying (18), and probiotic/fermented foods improve digestion, increase bioavailability of nutrients, and enhance immunity (8, 19–23). Yogurt can also serve as a vehicle for the consumption of other healthful foods such as cereals, nuts and fruits to form a complete meal, thus also improving overall diet quality (24). Due to its potential benefits and unique characteristics, GY warrants further investigation.

Greek Yogurt (GY), also draws attention as a potential post-exercise health food due to its high protein content (mostly casein) which is created during the manufacturing and condensing process in which GY is made from regular yogurt (25). To date, only regular yogurt but not GY has been studied in combination with exercise for strength, muscle and body composition. One study in young, normal weight, untrained females indicated no further benefit of regular yogurt (5 g protein/serving, 3x/day) plus RT on increasing strength and lean mass compared to a protein-matched control and a CHO control (26). In addition, only 2 studies using regular yogurt (5 g protein/serving, 2–3 servings/day) in a weight loss context have been conducted, and results on body composition were inconclusive (23). These studies were conducted in overweight, predominantly female populations, and only one included an exercise (RT) component (27, 28). In most of these studies, the amount of protein provided by the regular yogurt [5 g × 3 servings per day (27) and 5 g × 2 servings per day (28)] was likely insufficient to enhance adaptive remodeling favoring the yogurt groups. Previous research in young individuals determined that an isolated dose of 20 g of protein was superior in stimulating MPS as compared to lower isolated doses of 5 and 10 g (29, 30). GY contains 3 to 4-fold the amount of protein as regular yogurt. One serving of plain GY (175 g) provides 17 g protein (31). The same amount of protein can be consumed from ~500 ml of milk (2 servings) (32). Given the effectiveness of milk in this context (12, 13) and noting the additional potential benefits of yogurt consumption (15, 33–35), interventions assessing similar effects on body composition, strength and other related health outcomes using GY are warranted.

Thus, the aim of our study was to assess whether the consumption of GY will increase strength and muscle thickness, and improve body composition more than a semi-solid, CHO-based placebo pudding (PP) following a 12-week exercise (RT and plyometric [PLY]) training intervention in untrained, university-aged males. Although both groups should experience favorable training adaptations, we hypothesized that GY supplementation would facilitate significantly greater increases in strength, muscle thickness, and fat free mass (FFM) while reducing fat mass (FM) compared to the PP group.

## Methods

### Participants

Thirty healthy, university-aged (18–25 y) males were recruited for the study from the Brock University (Ontario, Canada) student population. Following a general screening protocol, subjects that were free of medical conditions were eligible to participate in the study. Screening ensured participants were untrained (RT <0–2 times/week for last 6 months), of normal body fatness (<25% fat), and had not been consuming dietary supplements (e.g., vitamins, minerals, protein supplements, creatine) in the last 6 months prior to entering the study. Once all inclusion criteria were met, subjects were informed of potential study risks, and written informed consent was obtained. The protocol was approved by the Brock University Research Ethics Board and conformed to all standards of Canada's Interagency Panel on Research Ethics for conducting human research.

### Supplement Protocol

This study was a parallel randomized controlled trial (clinical trial registration #: NCT03196856). Subjects were randomized to one of two groups; GY group (GY; *n* = 15) or placebo pudding group (PP; *n* = 15). Participants randomized to the GY group consumed 200 g of Oikos 0% fat, plain GY (~110 Kcals, 20 g protein, 8 g CHO; Danone Canada Inc., Boucherville, Quebec) 3 times/day on training days (immediately post-exercise, 1 h post-exercise and before bed) and 150 g, 2 times/day on non-training days (breakfast and before bed). To encourage compliance, participants could flavor the GY with calorie-free sweeteners or syrups if they preferred. The control group consumed 47 g of a placebo pudding (PP), which was an isoenergetic, chocolate flavored, CHO-based semi-solid food (~110 Kcals, 0 g protein, 28 g CHO) on the same supplement schedule as the GY group. The PP was comprised of maltodextrin (2 parts), chocolate pudding powder (1 part), and water, and was designed to resemble the consistency and texture of GY. The PP was made by the same researcher during the entire duration of the study. To ensure anonymity of the PP, it was termed the “*study-designed supplement”* and its contents were kept discreet to participants and other study personnel (e.g., exercise trainers). In fact, many subjects (and trainers) within the PP group believed that this supplement was the “test product” and that it may have contained muscle-supporting nutrients such as protein. Both groups had their respective supplements divided into individual serving containers and labeled by study personnel. On training days, the post-exercise doses were consumed in the research lab following training with study personnel present, whereas on non-training days and before bed, doses were consumed away from the laboratory and/or at home. These supplements were given to the participants to take home on a weekly basis. During the study, both groups were encouraged to maintain their habitual diets, except for the intervention food. Participants were provided with the same information and advice to help them compensate for the added calories consumed from the supplements.

### Training Protocol

Both intervention groups underwent 12 weeks of exercise training, 3 days/week, at the campus gym or in other equipped research laboratories at Brock University. All training was facilitated by certified trainers and/or trained senior kinesiology students to ensure proper lifting form and to provide motivation to the subjects. Each formal training session (~60 min) consisted of either full-body RT (2 d/week) which included exercises such as leg press, bench press and seated row (at ~2% 1-RM, 8–10 total exercises, 3–4 sets/exercise, 8–12 reps/set), or PLY training (1 d/week) which included exercises such as box jumps and frog jumps (150–250 total jumps/impacts per session). The training sessions followed the principles of undulated periodization (36), varying intensity and/or volume throughout the intervention. Within this training paradigm, most RT exercises were taken to voluntary failure (or close).

### Dietary Analysis

Participants recorded their habitual food and drink intake prior to beginning the study and again during the 12th week of training using a 7 and 3-days food diary, respectively. The 3-days food diary consisted of 2 weekdays and 1 weekend day. Instructions on how to fill out a food diary were thoroughly explained to each participant in advance. Upon completion, food diaries were examined, and any uncertainties were clarified with the subject by the research personnel. Dietary intake was inputted and analyzed using a diet analysis program (Food Processor, ESHA Inc., Salem, OR). All food diaries were inputted and analyzed by the same researcher.

### Strength Assessment

Muscular strength was evaluated via voluntary 1 repetition maximum (1-RM) testing of four exercises at baseline and following week 12 of the intervention. Participants arrived hydrated and fed on testing days and did not participate in any structured exercise for a minimum of 48 h prior to testing. 1-RMs were determined for the following exercises: chest press, seated row, leg extension, and hamstring curl. Participants were made familiar with the exercises and testing protocol by doing light (estimated 40–50% 1-RM), practice repetitions (8–12) before the actual assessments began. During the assessment, weight was progressively added to each exercise until 1-RM was determined. Rests of 2–3 min were given between each set. Failure was determined when participants were unable to complete the full range of motion of a repetition without compensation. If 1-RM was not determined after 4 consecutive sets, it was estimated using the O'Connor calculation [1-RM = weight × (1+ (0.025 × reps))] (37, 38) from the set with the lowest number of completed reps. The use of a predictive equation for estimating 1-RM has been previously validated in a young, untrained male population (38, 39). Weight was adjusted so that most participants experienced voluntary failure at 4 or less repetitions. All pre and post-testing for all outcome variables (strength, muscle thickness, and body composition) were completed by the same researcher.

### Muscle Thickness Assessment

Muscle thickness was measured via ultrasonography (GE Medical Systems, Ultrasound Vivid I portable, Milwaukee, WI, USA.). Muscle thickness was measured at 2 locations: the biceps brachii and the quadriceps femoris (rectus femoris + vastus intermedius). Muscle thickness for the quadriceps was measured at 50% between the greater trochanter and lateral epicondyle of the femur. For the biceps, muscle size was measured at 40% from the proximal end between the greater tubercle and the lateral epicondyle of the humerus. These sites correspond to where the muscle belly is the thickest. Subjects laid in a supine position, relaxed, with palms facing into their body. A thin layer of gel was applied to each muscle site and the ultrasound probe was placed on the site without depressing the skin. The measurement was obtained by pressing the probe gently on skin and moving it over the muscle. Muscle thickness was measured from the bone to the outer/superficial sarcolemma. Three images were obtained for each site and then averaged to obtain a final value. Ultrasound tests were completed 48–72 h following exercise. All testing occurred in the morning, with subjects fasted (10–12 h).

### Body Composition Assessment

Body composition was assessed using air-displacement plethysmography via the bod pod (COSMED USA Inc., BODPOD, Chicago, Il.). On testing days, subjects arrived at the laboratory in the morning, fasted (10–12 h), changed into compression shorts and put on a swim cap (the same outfit for each participant was used for the pre- and post-intervention measures). The testing procedure began with calibration of the empty chamber with a known volume. Participants then stepped into the bod pod and sat inside the unit for 45 s where their raw body volume is determined as the volume of air displaced (the difference between the volume of the empty vessel and the volume of the vessel with the participant inside). Body volume was entered into a pre-set equation accounting for body weight (measured on a scale before they entered the bod pod), height (measured on a stadiometer before they entered the bod pod), age, and ethnicity. Thoracic volume was predicted based on the Siri density model. Two tests were completed for each participant and compared, if the variation between the two tests was large (as assessed directly by the bod pod), a third test was completed. FFM, FM, and % body fat were then estimated via calculations.

### Statistics

Data were analyzed using SPSS (IBM Corp. Released 2013. IBM SPSS Statistics for Windows, Version 22.0. Armonk, NY: IBM Corp.). Data were checked, and normality was confirmed by assessing measures of central tendency and homogeneity of variances, and sphericity. Data points that were more than +/−2 SD from the mean were categorized as outliers and removed. Missing data points (1 GY participant for all post-strength measures, 1 GY and 1 PP participant for post-ultrasound measures, 2 GY participants and 1 PP participant for post-dietary data) were replaced with the series mean for that timepoint. Repeated measures ANOVA (RMANOVA) was used to analyze time (pre and post), intervention (GY vs. PP), and interaction effects (intervention x time). Independent *t*-tests were used to analyze baseline data and percent change data between the groups. An ANCOVA design was used to assess changes over time while controlling for baseline % body fat differences.

## Results

### Baseline Characteristics

Thirty participants were randomized, and 27 participants completed 12 weeks of the intervention. One GY participant stopped exercise after 6 weeks due to injury (unrelated to the study). Two participants (1 PP and 1 GY) ended the study early (after 6 weeks of training) because they moved away from the university area. Post-testing was completed on all three of these subjects and their data were included in the analysis, except for the injured participant who did not complete the 1-RM post-testing.

There were no differences at baseline for any variable between groups except for % body fat, with PP being higher than GY (*p* = 0.049). This was also reflected in the fat mass measure although the difference did not reach statistical significance (*p* = 0.066).

### Adherence to the Study Supplements and Exercise Program

Trainers and study personnel ensured that the post-exercise supplement doses (of either GY or PP) were consumed in the laboratory, following training. This produced a 100% adherence rate for the post-exercise supplements. Bedtime and non-training day supplement doses were consumed without direct supervision. Food diaries completed at week 12 indicated a 97 and 99% adherence rate for the intake of the unsupervised supplements for the GY and PP groups, respectively. Training was well-tolerated, and attendance was 31.6 and 30.1 out of 36 sessions for the GY and PP groups equating to an 88 and 84% adherence rate, respectively, which was not significantly different between groups.

### Strength (Table 1; Figure 1)

There was a significant main time effect for all 1-RM strength exercises (*p* < 0.001). Significant interaction effects for the chest press (*p* = 0.026), seated row (*p* < 0.001), leg extension (*p* = 0.004), and 1-RM total (*p* < 0.001) indicated that the GY group gained more strength over time for these exercises than the PP group.

**Table 1:** 1-RM Strength measurements pre- and post-training.

	**Greek yogurt**				**Placebo pudding**				**RM-ANOVA**		
	***n***	**Pre**	**Post**	**Change**	***n***	**Pre**	**Post**	**Change**	**Time**	**Group**	**Interaction**
		**kg**	**kg**	**Δ**		**kg**	**kg**	**Δ**	***p*-value**	***p*-value**	***p*-value**
Chest press	14	81 ± 23	103 ± 20	22 [13.1–24.6]	15	87 ± 18	100 ± 20	13 [9.3, 16.9]	<0.001	0.82	0.026
Seated row	15	84 ± 21	105 ± 23	21 [15.1, 23.5]	15	83 ± 17	93 ± 17	10 [6.9, 16.9]	<0.001	0.34	<0.001
Leg extension	15	111 ± 24	150 ± 21	39 [29.4, 45.1]	15	124 ± 22	148 ± 27	24 [21.1, 30.7]	<0.001	0.51	0.004
Leg curl	15	79 ± 16	92 ± 15	13 [7.3, 14.8]	15	85 ± 15	94 ± 17	9 [6.1, 14.8]	<0.001	0.42	0.22
1-RM total	15	357 ± 80	455 ± 79	98 [72.6, 110.6]	15	379 ± 67	435 ± 76	57 [48, 65.3]	<0.001	0.96	<0.001

*Strength values (absolute values displayed as mean ± SD, change values displayed as mean [95% CI]). Statistical analysis was by RM-ANOVA with time (pre and post) as the within factor and group (GY and PP) as the between factor. Significance was set at p < 0.05*.

**Figure 1:**
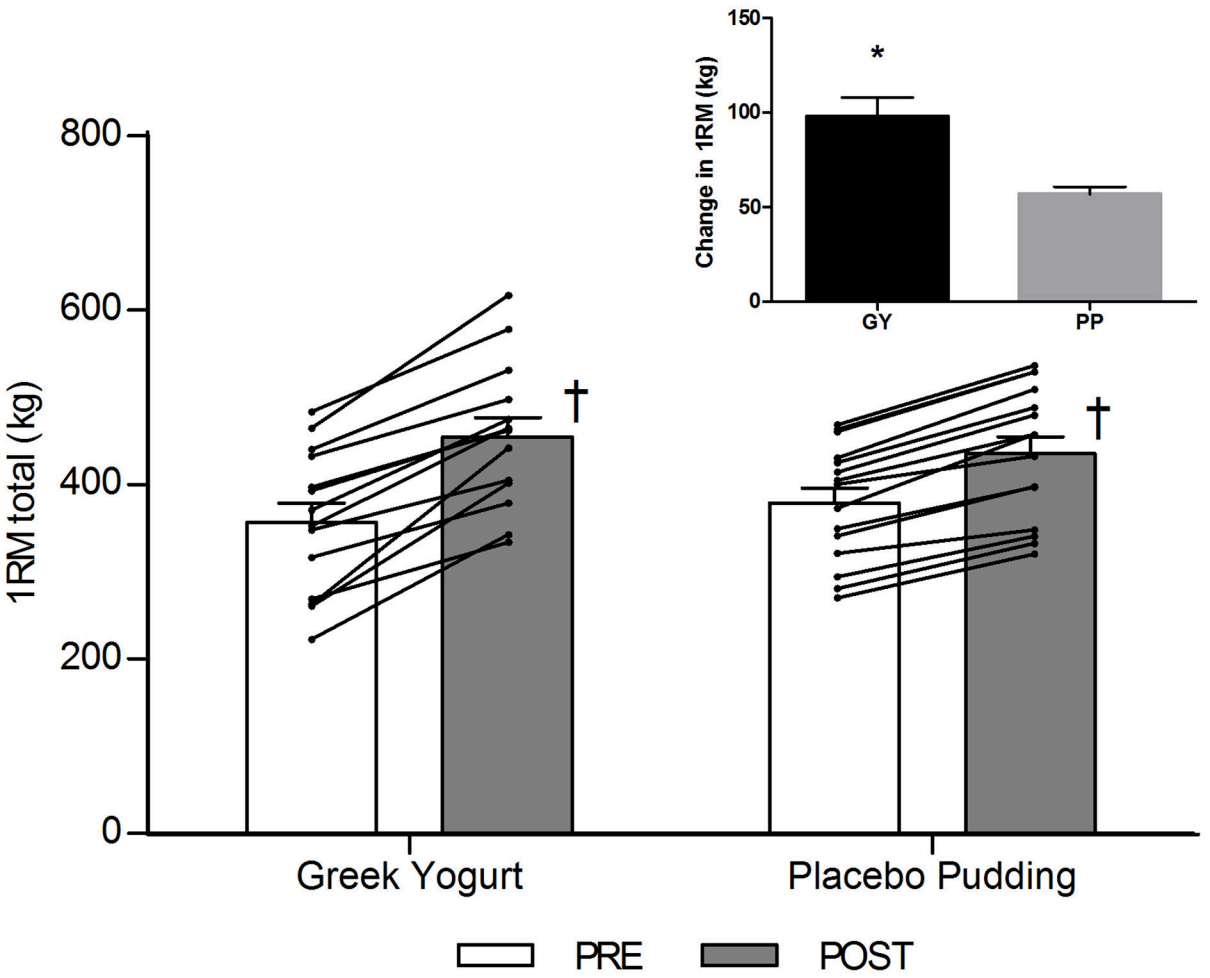
Total 1-RM strength before and 12 weeks after RT and PLY in GY (*n* = 14) and PP (*n* = 15) groups. Individual pre and post-responses are represented by the lines over the bars. The inset graph shows the change in total 1-RM strength from baseline. ^†^Significantly different from Pre within the same group (*p* < 0.05). *Significantly different from PP in the change from baseline in inset (*p* < 0.001). Values are presented as mean ± SE. RM = Repetition maximum.

### Muscle Thickness (Table 2)

Main time effects were present for muscle thickness of the biceps and the quadriceps (*p* < 0.001). A significant interaction effect for muscle thickness of the biceps indicated that the GY group increased their average muscle thickness to a greater extent compared to the PP group (*p* = 0.004). Intra-operator variability (%CV) was 0.94% (95% CI = 0.5, 1.38) and 0.83% (95% CI = 0.6, 1.05) for biceps and quadriceps measures of muscle thickness, respectively. The change in biceps thickness correlated with the change in total strength across all participants (*R* = 0.61, *P* = 0.001).

**Table 2:** Muscle thickness measurements analyzed using ultrasonography of the biceps and quadriceps muscles pre- and post-training.

	**Greek yogurt**				**Placebo pudding**				**RM-ANOVA**		
	***n***	**Pre**	**Post**	**Change**	***n***	**Pre**	**Post**	**Change**	**Time**	**Group**	**Interaction**
		**cm**	**cm**	**Δ**		**cm**	**cm**	**Δ**	***p*-value**	***p*-value**	***p*-value**
Biceps	13	2.64 ± 0.4	3.1 ± 0.4	0.46 [0.23, 0.51]	14	2.75 ± 0.4	2.87 ± 0.5	0.12 [0.01, 0.25]	<0.001	0.70	0.004
Quadriceps	14	3.81 ± 0.8	4.47 ± 0.8	0.66 [0.34, 0.8]	14	3.65 ± 0.7	4.06 ± 0.7	0.41 [0.21, 0.65]	<0.001	0.27	0.14

*Muscle thickness values (absolute values displayed as mean ± SD, change values displayed as mean [95% CI]). Statistical analysis was by RM-ANOVA with time (pre and post) as the within factor and group (GY and PP) as the between factor. Significance was set at p < 0.05*.

### Body Composition (Table 3; Figures 2–4)

A significant main effect of time was observed for FFM (*p* < 0.001). A significant interaction effect for FFM indicated that the GY group increased FFM more than the PP group (*p* = 0.046). There was a significant main effect of group for FM (*p* = 0.035), with GY subjects having a lower FM than PP subjects regardless of timepoint. There was a significant main effect of group for % body fat (*p* = 0.022), with GY subjects having less % body fat than PP subjects. Because there was a significant difference in % body fat between groups at baseline, an ANCOVA was used with baseline % body fat as a covariate, to assess the change in % body fat between groups. The ANCOVA indicated that the GY group reduced % body fat significantly more than the PP group (*p* = 0.048). **Figure 4** expresses the mean lean and fat mass changes as a percent of the total weight change over 12 weeks per group. The GY group appears to have a more favorable body composition change (i.e., increase in FFM and decrease in FM) than the PP group. That is, all body mass the GY group gained was FFM and they lost fat mass (100 and −26%, respectively), whereas the PP group gained both FFM and fat mass (76 and 24%, respectively).

**Table 3:** Body composition measurements as assessed by Bod Pod pre- and post-training.

	**Greek yogurt**				**Placebo pudding**				**RM-ANOVA**		
	***n***	**Pre**	**Post**	**Change**	***n***	**Pre**	**Post**	**Change**	**Time**	**Group**	**Interaction**
				**Δ**				**Δ**	***p*-value**	***p*-value**	***p*-value**
Body mass (kg)	14	69.9 ± 9.6	71.8 ± 9.5	1.9 [0.3, 3.1]	15	69.7 ± 10.4	71.4 ± 10.4	1.7 [0.4, 2.3]	<0.001	0.935	0.776
Fat-free mass (kg)	14	60.1 ± 7.9	62.5 ± 7.6	2.4 [1.5, 3.2]	15	57.5 ± 6.9	58.8 ± 6.5	1.3 [0.5, 2]	<0.001	0.25	0.046
Fat mass (kg)	14	8.6 ± 4.0	8.1 ± 4.4	−0.5 [−1.4, 0.6]	15	12.2 ± 6.0	12.6 ± 5.4	0.4 [−0.9, 1.6]	0.918	0.035	0.296
Body fat (%)	14	12.3 ± 4.5	11.2 ± 5.1	−1.1 [2.2, 0.2]	15	16.9 ± 7.2	17.0 ± 6.1	0.1 [1.3, 1.6]	0.35	0.022	0.205

*Body composition values (absolute values displayed as mean ± SD, change values displayed as mean [95% CI]). Statistical analysis was by RM-ANOVA with time (pre and post) as the within factor and group (GY and PP) as the between factor. Significance was set at p < 0.05*.

**Figure 2:**
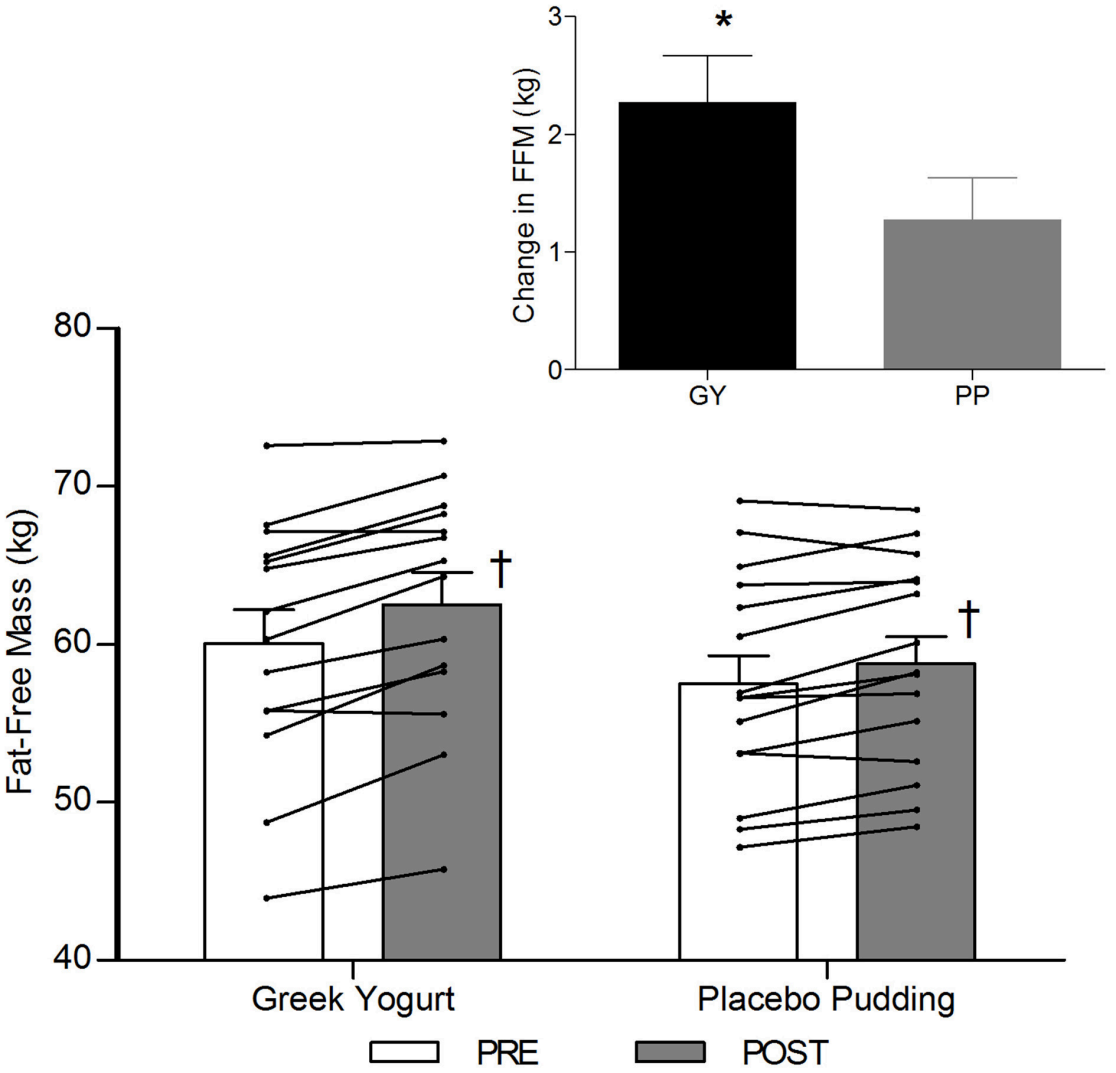
Fat-free mass before and 12 weeks after RT and PLY in GY (*n* = 14) and PP (*n* = 15). Individual pre and post-responses are represented by the lines over the bars. The inset graph shows the change in total fat-free mass from baseline. ^†^Significantly different from Pre within the same group (*p* < 0.05). *Significantly different from PP in the change from baseline in inset (*p* < 0.05). Bars are presented as mean ± SE.

### Nutrition (Table 4)

Main time effects were present for energy (*p* = 0.022), protein (absolute and relative to body weight; *p* < 0.001), carbohydrate (absolute; *p* = 0.003, and relative; *p* = 0.009), and calcium (*p* = 0.007) intake throughout the intervention. Significant Interactions for protein intake (absolute and relative) and calcium intake indicated that the GY group had greater intakes than the PP group (<0.001). A significant interaction for carbohydrate intake (absolute and relative) indicated that the PP group had greater intakes than the GY group (*p* = 0.002). There were no significant differences in fat intake throughout the intervention.

**Table 4:** Total daily nutrient intakes from food diaries for each group, at baseline and week 12.

**Nutrient intake per day**	**Greek yogurt**			**Placebo pudding**			**RM-ANOVA**		
	***n***	**Baseline**	**Week 12**	***n***	**Baseline**	**Week 12**	**Time**	**Group**	**Interaction *p*-value**
Energy (kcal)	14	2146 ± 407	2207 ± 345	15	1989 ± 398	2303 ± 588	0.022	0.83	0.11
Protein (g)	13	90.6 ± 15.2	124.8 ± 13.4	15	85.7 ± 14.6	85.9 ± 19.9	<0.001	<0.001	<0.001
Protein (g/Kg)	13	1.31 ± 0.32	1.74 ± 0.31	15	1.25 ± 0.26	1.22 ± 0.27	<0.001	0.007	<0.001
CHO (g)	15	246.1 ± 52.2	242.2 ± 55.2	14	225.0 ± 54.9	283.3 ± 55.2	0.006	0.57	0.002
CHO (g/Kg)	13	3.46 ± 0.87	3.38 ± 0.71	14	3.3 ± 0.89	4.04 ± 0.9	0.013	0.416	0.002
Fat (g)	15	79.2 ± 18.0	78.4 ± 18.6	15	79.9 ± 27.5	84.9 ± 35.7	0.57	0.68	0.43
Fat (g/Kg)	15	1.18 ± 0.27	1.11 ± 0.26	15	1.15 ± 0.37	1.19 ± 0.46	0.81	0.84	0.32
Calcium (mg)	14	699 ± 267	1069 ± 243	14	678 ± 225	585 ± 211	0.007	0.003	<0.001

*Nutrient intake values (displayed as mean ± SD). Nutrient intakes include daily diet and GY or PP supplementation. Statistical analysis was by RM-ANOVA with time as the within factor (week 0 and week 12) and group as the between factor. Significance was set at p < 0.05*.

### Percent Change Analyses (Table 5)

Percent change was calculated for each variable using the equation: [(post-pre)/pre) × 100]. Independent *T*-Tests revealed a greater percent change decrease in FM and % body fat in the GY group compared to the PP group (*p* = 0.042 and *p* = 0.038, respectively). Similar to the RM-ANOVA results, percent change for the biceps muscle thickness and 1-RM strength measures (except the leg curl) were greater for the GY group compared to the PP group.

**Table 5:** Percent (%) change for both groups, from pre to post-intervention.

**Variable**	**Greek yogurt**		**Placebo pudding**		**Independent *T*-test**
	***n***	**%**	***n***	**%**	***p*-value**
Body mass (Kg)	15	2.4	14	2.0	0.77
Fat-free mass (Kg)	15	3.9	15	2.3	0.11
Fat mass (Kg)	14	−11.1	14	5.8	0.042
Body fat (%)	14	−13.2	13	−1.1	0.038
Biceps muscle thickness (cm)	14	16.4	13	7.1	0.026
Quadriceps muscle thickness (cm)	14	15.0	14	13.0	0.67
Chest press (Kg)	13	28.3	15	15.4	0.030
Seated row (Kg)	13	23.7	14	11.7	0.002
Leg extension (Kg)	14	11.7	14	20.9	0.006
Leg curl (Kg)	13	14.6	14	12.8	0.62
1-RM total (Kg)	13	26.8	15	15.1	0.003

*% change values (displayed as means). Statistical analysis was by independent t-test between groups (GY and PP). Significance was set at p < 0.05*.

## Discussion

Our data demonstrate that the consumption of plain 0% fat GY (600 g on training days, 300 g on non-training days) following resistance and plyometric exercise as part of a 12-week training program increased most measures of strength, biceps muscle thickness and fat free mass while reducing FM more than an isoenergetic, CHO-based placebo pudding consumed at the same timepoints. This study is the first to use GY in this context and demonstrate such an effect with resistance exercise.

Strength was one of our main outcome measures. Strength is an important functional measure and can be used as a surrogate for muscle size and lean mass as these variables are highly correlated (40). Although strength increased in both groups following the intervention, our data revealed a significant time by group interaction effect for the chest press, seated row, and leg extension exercises as well as the composite measure of 1-RM total (Table 1; Figure 1), indicating that the GY group increased strength more than the PP group. Our research supports previous findings in young, untrained adults where milk and RT was shown to increase strength greater than a CHO placebo (12, 13). However, some training studies that utilize different whole dairy foods like chocolate milk (17) or regular yogurt (26) showed no additional strength increases compared to a CHO placebo. This may be because the amount of protein provided in the aforementioned studies was insufficient to see divergent strength adaptations between the groups. For example, both dairy groups, after supplementation, were habitually consuming only 1.3 g/kg/day (17) and 1.0 g/kg/day (26) of protein, which not only was close to the amount of protein consumed by their respective placebo groups, but also below the recommended threshold of protein intake for novice exercisers. Research suggests protein intakes of approximately 1.6 g/kg/day are necessary for individuals new to RT to facilitate optimal strength adaptations (2, 41, 42). The justification for the increased protein recommendation of 1.6 g/kg/day is due to a higher rate of MPS in novices (43) and a reduced efficiency of protein utilization compared to trained individuals (44). In our study, GY supplementation enabled subjects to increase their protein intake to 1.74 g/kg/day (whereas PP subjects consumed 1.22 g/kg/day of protein). This could explain why significantly greater strength gains were observed in this group. Our research is consistent with other chronic (minimum 10 weeks) training studies in young, untrained males which demonstrate that increased protein intakes optimize strength adaptations during a RT program (12, 45–47).

Initially, during a RT program strength gains are typically the result of neurological adaptations (48). However, to continue to develop muscular strength, morphological adaptations are necessary. These adaptations include increasing muscle cross sectional area (CSA) by increasing contractile proteins, altering tendon and connective tissue, changes in fiber type and hyperplasia, all of which require additional dietary protein (48). RT causes metabolic and mechanical stress to the muscle which signals MPS (49, 50). Once this stimulus has occurred, hyperaminoacidemia is required to facilitate the incorporation of amino acids (AAs) into the muscle to make new myofibrillar proteins (51). If this process is consistently repeated, like our study design intended, total muscle CSA can increase (52). Therefore, another outcome measure in our study was muscle thickness, a surrogate for muscle CSA, via ultrasonography (53). Ultrasonography has been shown to be reliable and valid for this measure when compared to MRI (54, 55). Our study revealed a significant main effect of time for biceps and quadriceps muscle thickness following the intervention (Table 2). This can likely be attributed to the effectiveness of the RT program in stimulating muscle hypertrophy (56). Our data also indicated that habitual consumption of GY yielded greater increases in biceps brachii muscle thickness compared to the consumption of the PP. These findings are supported by similar research in milk (12, 16) and isolated dairy proteins (e.g., whey and casein) (45, 47, 57) where greater increases in muscle size were seen in these groups compared to a placebo following RT in untrained, young, adult subjects.

**Figure 3:**
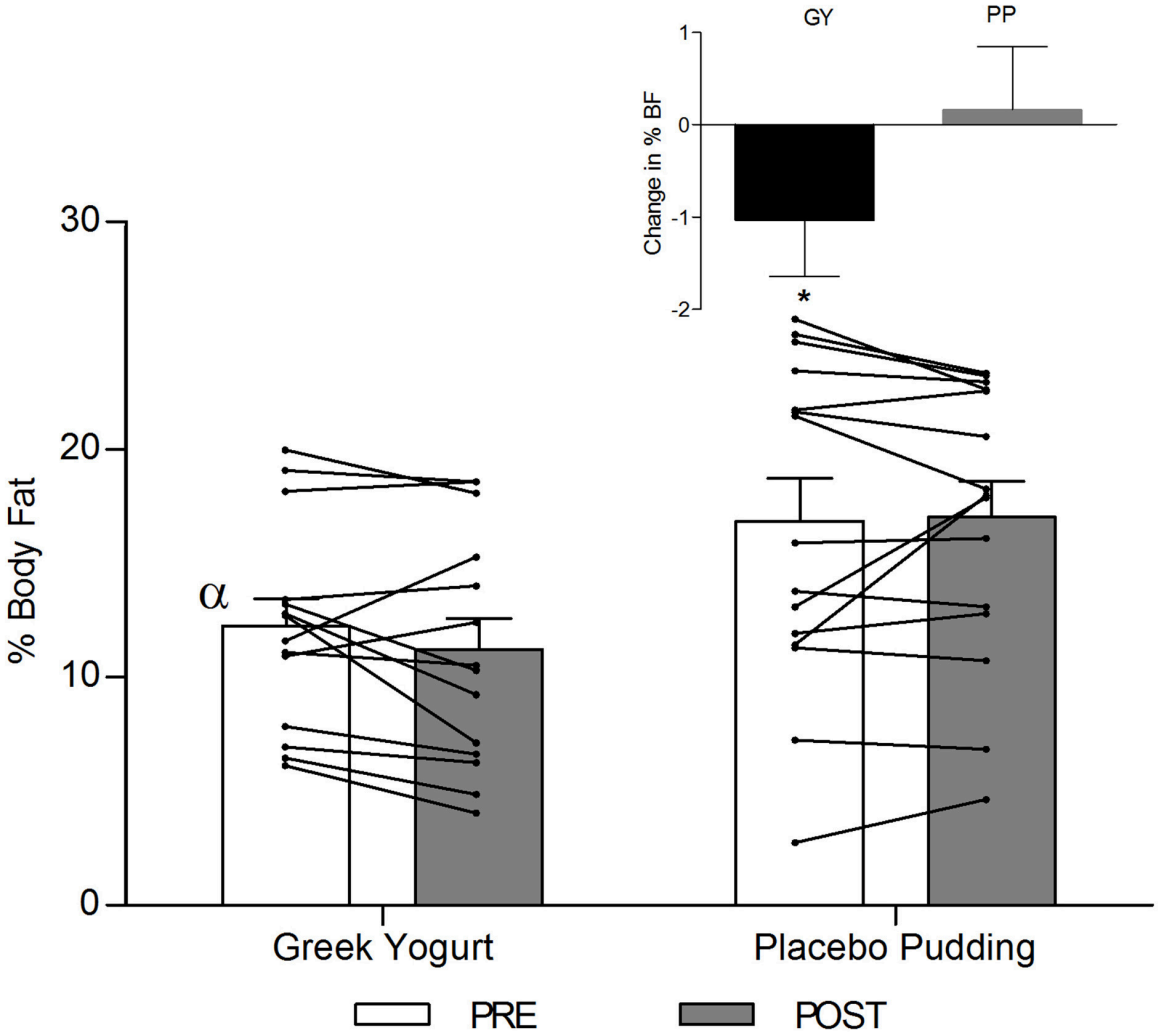
Total fat-free mass before and 12 weeks after RT and PLY in GY (*n* = 14) and PP (*n* = 15). Individual pre and post-responses are represented by the lines over the bars. The inset graph shows the change in total fat-free mass from baseline. α Significantly different at baseline between groups (*p* < 0.05). *Significantly different from PP in the change from baseline in inset as assessed by ANCOVA (*p* < 0.05). Bars are presented as mean ± SE.

**Figure 4:**
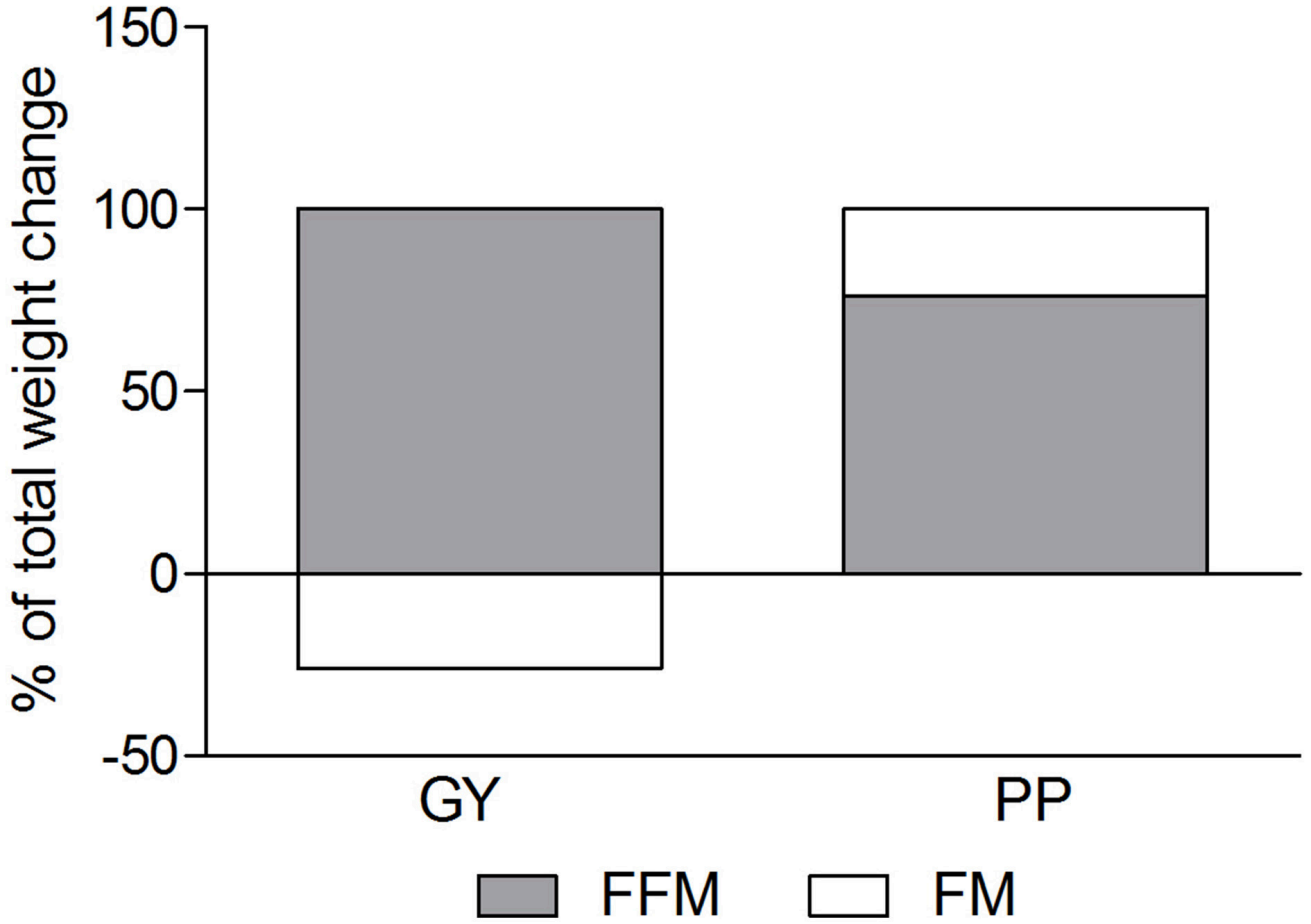
Fat mass and Fat-free mass visually expressed as a percent of total mass change during the intervention for the GY and PP groups (GY = 14, PP = 15).

Our data also demonstrated a favorable change in body composition for the GY group compared to the PP group. GY subjects gained significantly more FFM and reduced FM and % fat greater than PP subjects (Figures 2, 3 and Tables 2, 5). Although both groups gained significantly more body mass, all the body mass gained in the GY group was FFM and they were able to lose FM (100 and −26%, respectively). Whereas, the PP group gained both FFM and FM (76 and 24%, respectively). This represents a more favorable body compositional change in the GY group (Figures 2–4). Reductions in fat mass and increases in lean mass with GY are likely also due to other characteristics and nutrients in GY aside from protein. GY, as a semi-solid food, is satiating (58, 59) which can reduce hunger and delay subsequent energy intake (60), and it contains calcium which has been shown to inhibit intracellular lipogenesis, promote lipolysis and increase lipid oxidation (7, 61).

The current study provided subjects with 2 × 20 g doses of dairy protein from GY within 1 h post-exercise. This was done to ensure protein was provided to the working muscles in close temporal proximity to exercise and was designed to mimic similar research done in milk (12, 13). Research in young adults indicates 20 g of protein is just as effective at stimulating MPS as 40 g (29, 30), and, expressed relative to body weight and per meal, that 0.24 g/kg/meal is sufficient to stimulate myofibrillar protein synthesis (62). A recent review by Schoenfeld and Aragon (42) proposed a greater protein dose to maximize anabolism of 0.4 g/kg/meal, especially when the protein source is slower digesting (potentially like semi-solid Greek yogurt) and when consumed in the presence of other macronutrients which may further delay AA absorption (42). Based on these two dosing recommendations, for a 70 kg male (like those in our study), this corresponds to 16.8 and 28 g of protein per bolus, respectively. Our dose was within these recommendations and thus likely contributed to the greater training adaptations incurred with GY over time. Although participants in our study experienced increases in muscle thickness, fat-free mass and strength, it is important to note that myofibrillar muscle protein synthesis (mMPS) was not directly measured. It, therefore, cannot be directly concluded that our intervention lead to increases in mMPS, nor that only muscle tissue changes are primarily responsible for the observed effects (63).

Our participants also consumed GY prior to sleep in efforts to attenuate the rise in MPB and maintain a positive net protein balance during sleep, since sleep tends to be a fasted period in which protein balance naturally favors breakdown (64, 65). Interestingly, an acute study demonstrated that casein supplementation (40 g) prior to sleep was able to increase blood AA levels during sleep (7.5 h) and significantly increase whole body protein synthesis and net protein balance vs. the placebo (66). A training study subsequently confirmed that 28 g of casein given prior to sleep as part of a 12 week RT program (3 times/week) produced greater muscle mass and strength gains compared to a non-caloric placebo (flavored water) (45). Since GY primarily consists of casein protein, a similar mechanism of inhibiting or attenuating MPB may have occurred in our study resulting in greater cumulative strength, size and FFM gains.

Compared to milk, GY likely has a greater ratio of casein to whey [based on the manufacturing process of removing the liquid-whey from GY (23, 67)], it is more acidic (68), and it exists in a semi-solid food matrix (18). All these factors could attenuate digestion and subsequent absorption rates such that GY would elevate blood-AA concentrations for a longer duration than milk. Despite the plausibility of this hypothesis, no research exists on the post-prandial absorption rate and plasma AA response of GY compared to other dairy products, like milk. Research with intrinsically labeled casein protein indicates that absorption is even slower when consumed in a whole food matrix vs. isolated casein (69, 70). This research also demonstrated that a higher proportion of labeled casein consumed from milk was incorporated into skeletal muscle than when consumed as isolated casein, suggesting that the presence of other nutrients within the whole food may positively influence the utilization of AAs by muscle tissue (70). For this reason, GY may be at least as, or even more, beneficial at promoting a positive protein balance than milk or casein alone. On the other hand, it may also be possible that the presence of other factors within the GY food matrix (that are not present in milk) or the fact that it is a solid food could act to suppress the release of AAs too much and inhibit them from providing a sufficient trigger for MPS. Nonetheless, we did see positive adaptations with GY, so it may be possible that GY (a predominantly casein-based, semi-solid dairy product containing different nutrient compounds) may act to promote muscle adaptations via different mechanisms. More research on this using yogurt and other dairy and whole foods needs to be done.

High protein GY has demonstrated the ability to reduce appetite and energy intake in subsequent meals compared to lower protein snacks and snack-skipping (8). The GY and PP supplementation in our study was energy-matched and provided each group with 330 calories per training day (3 doses of supplement) and 165 calories per non-training day (1.5 doses of supplement). Our data show that both groups did not completely compensate their habitual diets for the added supplementation which caused them to significantly increase their energy intake from baseline. The GY group only increased their habitual energy intake, on average, by 61 calories at week 12, compared to 314 calories in the PP group. Although this increase in energy was not statistically different between the groups, the consumption of 300+ kcals/day over time is arguably more physiologically significant and can lead to an increase in fat mass compared to an increase of 61 kcals/day (71). For example, a 6 month study that replaced caloric beverages with non-caloric beverages, a straight-forward strategy to reduce energy intake, resulted in 2.5% weight loss (71). This likely contributed to the PP group gaining fat mass, and, on the other hand, the GY may have been more satiating resulting in less of an increase in energy intake in this group.

Our study had several strengths. The use of only one tester for all subject pre- and post-testing was a strength that minimized inter-tester variation. All supplementation was prepared by the same individual to ensure consistency. We kept the contents of the PP discreet and called it the “study-designed supplement,” which we believe may have facilitated our high supplement adherence rates (because the participants may have thought the PP contained different beneficial bioactives). Also, trainers involved in the study were unaware of the contents of the PP to prevent bias. Our study also had limitations. Subjects were not blinded to which supplement group they were in. Blinding is notoriously difficult to achieve in nutrition studies (72–74). However, we did conceal the contents of the PP from our subjects (and trainers). We also did not use state-of-the-art measurement tools, such as DXA or MRI, for body composition determination (75–77). We used the Bod Pod which is unable to give a specific measure of muscle mass. However, the Bod Pod is considered a reliable method for measuring body fat in normal weight populations compared to DXA (78, 79). Lastly, since subjects were initially untrained, they may have experienced a learning effect on the 1-RM exercises which may partly explain the increased strength during the post-testing. However, this would have been consistent for all participants regardless of group and cannot explain the divergent results in favor of GY.

In summary, the consumption of fat-free, plain GY during a 12-week exercise program promoted greater improvements in strength, muscle thickness and body composition than a CHO pudding placebo in untrained, university-aged males. Our study is the first to report a positive effect of GY with exercise on a comprehensive set of muscle-related outcome variables, which allows us to robustly assert GY's favorable role within this context. Thus, given our specific results, GY should be considered as a viable post-exercise, whole food, protein source for individuals beginning a RT program with the goal of increasing strength and lean mass and decreasing fat mass. Furthermore, the beneficial characteristics of GY beyond protein, such as its satiating effect, probiotic cultures and micronutrient content may offer additional benefits, above other dairy products, to digestive (80–82) and bone (83–85) health, and may have further implications in different age groups including the elderly. Future research is needed to elucidate the multiple health effects of GY as part of a healthy diet (with or without exercise) that extend beyond muscular benefit in different contexts.

## Ethics Statement

This research study was approved by the Brock University Biosciences Research Ethics Board (BREB). Brock University, 1812 Sir Isaac Brock Way St. Catharines, Ontario, L2S 3A1, (905) 688-5550 x 3035.

## Author Contributions

AJ conceptualized the idea. AB and AJ designed research project with critical input from BR and WW. AB, HS, JB, and MN provided a critical role in data collection and analysis. AB and AJ carried out the statistical analysis. AB wrote initial version of the manuscript. All authors contributed to the final version of the manuscript.

### Conflict of Interest Statement

The authors declare that the research was conducted in the absence of any commercial or financial relationships that could be construed as a potential conflict of interest.
